# Insights into calcific aortic valve stenosis: a comprehensive overview of the disease and advancing treatment strategies

**DOI:** 10.1097/MS9.0000000000002106

**Published:** 2024-04-29

**Authors:** Hritvik Jain, Aman Goyal, Abeer T.M.A. Khan, Noor U. Khan, Jyoti Jain, Shrey Chopra, Samia A. Sulaiman, Murali Mohan Reddy, Kush Patel, Kaarvi Khullar, Mohamed Daoud, Amir H. Sohail

**Affiliations:** a Department of Internal Medicine, All India Institute of Medical Sciences (AIIMS), Jodhpur; b Department of Internal Medicine, Seth Gordhandas Sunderdas (GS) Medical College and King Edward Memorial (KEM) Hospital, Mumbai; c Department of Internal Medicine, Kasturba Medical College, Mangalore; d Department of Internal Medicine, Baroda Medical College, Gujarat; e Department of Internal Medicine, University College of Medical Sciences and Guru Teg Bahadur Hospital, Delhi; f Department of Internal Medicine, Government Medical College and Hospital, Gondia, Maharashtra, India; g Rawal Institute of Health Sciences; h Department of Public Health, Health Services Academy, Islamabad, Pakistan; i School of Medicine, University of Jordan, Amman, Jordan; j Department of Internal Medicine, Bogomolets National Medical University, Kyiv, Ukraine; k Department of Surgery, University of New Mexico Health Sciences, Albuquerque, New Mexico, USA

**Keywords:** aortic valve stenosis, aortic valve, emerging therapeutics, transcatheter aortic valve replacement

## Abstract

Aortic valve stenosis is a disease characterized by thickening and narrowing of the aortic valve (AV), most commonly due to calcification, which leads to left ventricular outflow obstruction called calcific aortic valve disease (CAVD). CAVD presents as a progressive clinical syndrome with cardiorespiratory symptoms, often with rapid deterioration. The modern-day pathophysiology of CAVD involves a complex interplay of genetic factors, chronic inflammation, lipid deposition, and valve calcification, with early CAVD stages resembling atherosclerosis. Various imaging modalities have been used to evaluate CAVD, with a recent trend of using advanced imaging to measure numerous AV parameters, such as peak jet velocity. Significant improvements in mortality have been achieved with transcatheter AV repair, but numerous therapeutics and modalities are being researched to delay the progression of CAVD. This article aims to provide a comprehensive review of CAVD, explore recent developments, and provide insights into future treatments with various novel modalities.

## Introduction

Highlights

- Ataciguat, eplerenone, pelacarsen, PCSK9 inhibitors, colchicine, denosumab, pioglitazone, folic acid, and alendronic acid are emerging drugs that show potential in the management and progression of calcific aortic valve stenosis (CAVS).
- Novel non-invasive ultrasound therapy may be an effective therapeutic modality for patients with CAVS.
- Extensive research is currently underway to develop reliable biomarkers that can effectively track the progression of CAVS in patients, given its association with increased mortality and complications such as stroke. Among these biomarkers are the T50 test, high-sensitivity cardiac troponin, growth/differentiation factor 15, ST2 protein, and microRNAs (miRNAs).

Aortic valve sclerosis is characterized by thickening of the aortic valve (AV), typically accompanied by mild calcification without a significant pressure gradient (defined as an aortic jet velocity <2 m/s)^1^. The clinical importance lies in the fact that it is the most frequent cause of left ventricular (LV) outflow obstruction in both children and adults^1^. This condition primarily arises from three underlying causes: a congenital valve abnormality, often with additional calcification (unicuspid or bicuspid valves), calcific disease affecting a trileaflet valve, and rheumatic heart disease (RHD).

As AV leaflet thickening and calcification progress, the increased stiffness of the valve leads to obstruction at the valve level and a transition from aortic sclerosis to aortic stenosis (AS)^2^, which is referred to as calcific aortic valvular disease (CAVD). Numerous well-documented complications associated with CAVD include heart failure, sudden cardiac death, pulmonary hypertension, arrhythmias, and an increased propensity for bleeding^3–10^. For asymptomatic patients, there are currently no established medical treatments proven to delay the advancement of valve leaflet disease^11^. Although earlier retrospective studies appear promising regarding the use of statin therapy, a recent large-scale randomized prospective study revealed that statin therapy does not effectively halt disease progression^12^. In a recent investigation conducted by Lee *et al*.^13^, it was discovered that dipeptidyl peptidase-4 (DPP-4) inhibitors possessing favorable pharmacokinetic and pharmacodynamic properties were linked to a reduced risk of AS progression. In this study of 212 patients, the occurrence of severe AS progression was less common in the group receiving favorable heart-to-plasma DPP-4 inhibitor concentrations (7.1%) compared to the unfavorable DPP-4 inhibitor concentrations group (29.0%; *P*=0.03) or the non-user group (29.6%; *P*=0.01). After accounting for age, baseline renal function, and AS severity in Cox regression analysis, the group with favorable concentration of DPP-4 inhibitors exhibited a significantly reduced risk of severe AS development [hazard ratio (HR) 0.116, 95% confidence interval (CI) 0.024 to 0.551, *P*=0.007]. According to the 2021 European Society of Cardiology (ESC)/European Association for Cardio-Thoracic Surgery (EACTS) guidelines for the management of valvular heart disease, the sole effective intervention for severe symptomatic AS is aortic valve replacement (AVR)^14^.

This review aims to shed light on promising emerging therapeutic modalities employed in the management of CAVD. Furthermore, to highlight the urgent need to integrate such novel modalities, our review comprehensively covers the epidemiology, pathobiology, clinical features, diagnostic modalities, current management guidelines, and prognosis of CAVD.

The current review also aims to provide physicians with an in-depth resource that ensures accurate diagnosis and management of patients, thus mitigating the risks of coronary artery and cardiovascular diseases in the context of CAVD, which are frequently associated with poor prognosis and increased morbidity and mortality rates.

## Methodology

We conducted a search in the PubMed/MEDLINE, EMBASE, Google Scholar, and Scopus databases from inception until October 2023. Keywords and medical subject heading (MeSH) terms included ‘Aortic valve disease,’ ‘calcific aortic stenosis,’ ‘senile aortic stenosis,’ ‘aortic valve calcinosis,’ and ‘calcification of aortic valve.’ Boolean operators ‘AND’ and ‘OR’ were utilized to combine these MeSH terms. Our study encompassed all randomized controlled trials, observational studies, narrative reviews, and case reports investigating CAVD. Covidence facilitated the reviewing process, with two independent reviewers (A.G. and S.A.S.) screening titles and abstracts of retrieved studies to determine relevance. Full texts of potentially relevant studies were obtained and further assessed for eligibility.

## Epidemiology

The reported incidence of CAVD has been found to vary between countries. In a study conducted in the year 2019, the highest number of reported cases has been reported in the United States (*n*=117 080), followed by Japan (*n*=71 837), which therefore increases concerns regarding the prevalence of CAVD^3^. According to the study, the prevalence of CAVD does not only vary amongst different nations but is also found to be notably higher among males and showed an upward trend with advancing age rather than females and younger age groups, accordingly^3^. In developed nations, after coronary artery disease and systemic arterial hypertension, CAVD is the third most common cardiovascular disease and affects the heart valves. According to a Norwegian study, there are 4.9 instances per 1000 people per year of CAVD cases. Projections show that due to present incidence rates and demographic changes, the number of patients with CAVD in wealthy countries over the age of 70 or 75 years will double or even triple in the next 50 years. However, because rheumatic fever (RF) and RHD are more common in developing nations, the epidemiology of AS differs^15^. In 2017, CAVD caused an estimated 12.6 million cases and 102 700 deaths worldwide. The disability-adjusted life years (DALYs) related to CAVD increased by 101% between 1990 and 2017. High-income areas, including Western Europe, the United States, Canada, Chile, Argentina, Australia, and New Zealand, had the highest death rates^16^.

While the prevalence of CAVD is 0.4% in the general population, it significantly increases to 1.7% in those over 65 years, with an estimated 3.4% prevalence of severe AS in people 75 years and older, frequently accompanied by symptoms^15^. Deaths attributed to CAVD have consistently increased over the past two decades, primarily affecting older adults in high-income countries. Children’s range from 0.5% to 1%. Nevertheless, it accounts for more than half of the CAVD cases, which necessitates surgical intervention. This contributes considerably to the AV issues. A noteworthy difference between those with bicuspid and tricuspid valves is that people with bicuspid valves frequently develop CAVD one to two decades earlier^15^. With a prevalence of 1841 per 100 000 individuals worldwide in 2017 for men aged at least 70 years, it is noteworthy that men are disproportionately afflicted^16^.

## Risk factors

Risk factors for AS include a sedentary lifestyle, obesity, dyslipidemia, DM, and hypertension. The risk of developing CAVD is enhanced by several risk factors such as metabolic syndrome, RF, and RHD. High blood pressure, obesity, and type 2 DM are modifiable risk factors. Intricate interactions between valve endothelial and interstitial cells, immune cells, and modifications to the hemostatic system, including platelet function, result in the development of CAVD. Risk evaluation is made more difficult by hemodynamic risk factors including reduced cardiac output and LV systolic dysfunction^17^.

Hereditary factors are believed to have an impact on regional variations in the prevalence of CAVD, which are characterized by clustering. The importance of signaling pathways, such as NOTCH, Wnt-catenin, and myocardin, in steering valvular cells toward a fibrocalcific lineage has been demonstrated by genetic and molecular research. The polygenic nature of CAVD has been demonstrated by ongoing genetic research, with a focus on lipid metabolism and cell signaling pathways connected to fibrosis, mineralization, and inflammation^15^.

Smoking and sedentary lifestyle are behavioral risk factors that have an impact on CAVD development. When age and sex were taken into account, a study indicated that sedentary activity elevated the risk of CAVD by 5.9 times, while hypertension, obesity, and DM were linked to 1.9, 1.8, and 1.6 times greater chances, respectively. These elements work together to create a complex environment for CAVD, making it a major public health concern^18^.

Cardiac risk factors have also been explored in multiple studies regarding their association with AS. Yan *et al*.^4^ examined a group of over 1.12 million Canadians in an observational study and followed them up for more than a decade, and they have demonstrated a significant association between common cardiovascular risk factors such as hypertension, DM, and dyslipidemia and the likelihood of developing severe AS. Compared with other risk factors, hypertension was found to have the highest attributed risk. The study also demonstrated that the impact of cardiac risk factors on the development of AS was consistent between females and males, although a higher incidence of AS was noted in the latter group^4^. The incidence rate of ischemic stroke was 13.3 per 1000 person-years in the control group, whereas it increased to 30.4 per 1000 person-years in patients with AV stenosis, indicating a significantly higher risk among patients with AV stenosis compared to the control group across all age groups^4^. Similarly, another study has also demonstrated that AS consistently poses an elevated risk of cardiovascular events^5^. Since there is a high prevalence and potentially poorer management of CAVD risk factors in countries with lower income, including hypertension, there could be a higher risk imposed on such nations.

## Molecular mechanisms of development

The etiology of AS has evolved significantly over the 20th century. Historically, RF was the main cause; however, in the modern world, AS is characterized by late-life onset and involves lipid deposition and inflammation in the AV. Genetic and environmental factors contribute to its development, with genome-wide association studies identifying key genetic polymorphisms related to factors such as lipoprotein metabolism, calcium signaling pathways, and genes such as RUNX2 and CACNA1C. Individuals with multiple affected siblings had a higher risk of AS, suggesting potential genetic factors. Mutations in NOTCH1 may also play a role, especially in families with a congenital bicuspid aortic valve (BAV)^19^.

CAVD has distinct initiation and propagation phases. The initiation phase shares similarities with atherosclerosis and involves endothelial damage, inflammation, and risk factors such as age, male sex, body mass index, smoking, hypertension, and elevated lipid levels. Mechanical stress plays a key role in the initiation of CAVD. The propagation phase is characterized by fibrosis, accelerated calcification, and a shift toward pro-osteogenic mechanisms. Factors such as reduced nitric oxide (NO) expression and upregulation of the renin–angiotensin system contribute to this phase. Valvular interstitial cells (VICs) undergo a phenotypic switch to osteoblast-like cells. Matrix Gla protein (MGP), a vitamin K-dependent protein, inhibits calcification, but vitamin K antagonist (VKA) drugs can exacerbate it^20^.

Recent research has identified genetic risk loci such as interleukin-6 (IL-6), ALPL, and NAV1 in CAVD. NAV1 exhibits a strong association between risk alleles and elevated NAV1 expression in the AV, which is associated with cardiovascular traits. IL-6 promotes valve mineralization, particularly through a specific SNP (rs1800795) in its promoter region. ALPL indirectly affects CAVD by influencing bone mineral density, whereas NAV1, linked to actin binding and expressed in the aorta, may play a role in vascular function^21^.

In AS and CAVD, modern-day pathophysiology involves a complex interplay of genetic factors, chronic inflammation, lipid deposition, and valve calcification. Early CAVD stages resemble atherosclerosis, with inflammation involving immune cell activation, leading to CAVD and obstruction of blood flow. Inflammation is driven by factors such as oxidized low-density lipoprotein (ox-LDL) and IL-6. Epigenetic factors and the microbiome may also contribute to this, and endothelial dysfunction is closely linked to calcific lesions. Lipid accumulation precedes inflammation, and lipoproteins (a) [Lp(a)] and oxidized phospholipids (OxPLs) contribute to inflammation and mineralization. Antioxidative endothelial cells release NO to inhibit valve interstitial cell differentiation into osteoblast-like cells; however, oxidative stress impairs NO production. Regulation of DPP-4 and insulin growth factor-1 (IGF-1) are implicated in calcification, and inhibition of BMP-2 and Notch signaling may offer therapeutic approaches. In CAVD, antigen-presenting cells (APCs), including macrophages and dendritic cells, play a pivotal role in inflammation. The imbalance between M1-like and M2 macrophages intensifies inflammation, whereas T lymphocytes, cytokines, and mechanical forces contribute to immune activation. NOTCH1 mutations are associated with CAVD pathogenesis, offering potential therapeutic targets along with the BGN-TLR3-IFNAR1 axis, which governs AV calcification^22–29^.

CAVD is a complex condition influenced by genetics and epigenetics. Genome-wide association studies have identified specific genetic variants linked to valvular calcification and AS, such as the LPA variant (rs10455872) and variants near PALMD, TEX41, GATA4, IL-6, and ALPL. Epigenetic modifications such as DNA methylation and long non-coding RNA H19 contribute to AV calcification via Notch-signaling pathways. Lp(a)-derived metabolites, such as lysophosphatidic acid, affect DNA methylation and PLPP3 activity, inducing pro-osteogenic effects. Multiomics has transformed CAVD research, with RNA sequencing (RNA-seq) revealing differential gene expression, especially in endothelia, and highlighting NOTCH1 haploinsufficiency networks. Proteomics identifies genes such as GFAP as specific VIC markers, whereas metabolomics unveils changes in metabolites and diverse valvular cell types. Research has explored the role of Runx2-expressing cells in CAVD and suggested potential therapeutic avenues. Oxidative stress is significant in CAVD and is driven by OxPLs that activate the NF-kB pathway and promote osteogenesis in VICs. Oxidative stress also reduces the NO levels and promotes osteogenesis. Asymmetric dimethylarginine, uric acid, and serotonin metabolism exacerbate oxidative stress. Oxidative stress is prominent in BAVs and affects disease progression. Capsaicin, with its anti-inflammatory and antioxidant properties, shows promise as a CAVD treatment. Studies on CAVD have identified differentially expressed genes, including circRNAs, lncRNAs, microRNAs (miRNAs), and mRNAs. Immune cell patterns revealed an increase in M0 macrophages and memory B cells as potential therapeutic targets. Research on rats has linked vascular calcification to chaperone genes and ER stress due to nicotine and vitamin D. Another study involving transcatheter aortic valve implantation (TAVI) in severe AS patients emphasized the role of microparticles in vascular health assessment^30–38^.

## Clinical features

### Classic clinical features

Most patients with CAVD are asymptomatic and are often discovered on diagnostic testing^39^. The initial presentation of the disease is often delayed until the valve orifice is narrowed to less than 1 cm^2^. Most patients with CAVD remain asymptomatic until the 7th to 8th decade of life in cases of CAVD as the hypertrophied LV can maintain cardiac output in the latent period. Anatomical abnormalities of the valve, such as a unicuspid or bicuspid AV, or the presence of metabolic diseases, such as alkaptonuria, can hasten the development of the disease and present in the 5th to 6th decades of life^40–42^. In symptomatic individuals, CAVD presents as a progressive clinical syndrome of cardiorespiratory symptoms with rapid deterioration. The cardinal features observed in cases of CAVD are angina pectoris, exertional dyspnea, and syncope^43^. Moreover, structural heart changes secondary to valvular obstruction can lead to the development of paroxysmal or recurrent arrhythmias that present as palpitations.

While severity is defined based on diagnostic tests, the onset of symptoms is associated with an overall poorer prognosis in patients with CAVD. Other late features of CAVD include heart failure, such as orthopnea, paroxysmal nocturnal dyspnea, and dyspnea due to pulmonary edema^44^. Symptoms of right heart failure and pulmonary hypertension may also develop in patients with AS; however, such cases are less common.

### Subclinical features

As most patients with calcific AS are asymptomatic, it is necessary to define the subclinical features of CAVD. The presence of AV calcification, as well as increased calcium in the AV identified on echocardiographic studies, has been linked to the development of structural changes in the heart, such as increased LV size and atrial dilatation^45^. These structural changes indicate that the presence of AV calcifications can serve as a precursor to the development of heart failure, which is often seen as the endpoint of CAVD. Even without the development of stenotic AVs, simple calcification of the AVs has been linked to a higher incidence of non-fatal myocardial infarctions and non-fatal cerebrovascular accidents^46^. Furthermore, new-onset cognitive impairment could be a presenting feature of subclinical AV calcification, indicating the effect of CAVD on the overall vasculature of the body^47^.

### Atypical presentations

Any deviation from the widely accepted natural course of the disease constitutes atypical presentation. While CAVD is generally present in the 7th or 8th decade of life, cases with much earlier presentations have been noted. These cases are generally associated with hereditary conditions or systemic inflammation, which hasten stenosis development. The unicuspid aortic valve is a rare entity that has been shown to present with AV calcifications as early as 3rd or 4th decade of life and is commonly misdiagnosed as a bicuspid aortic valvular disease^48^. Homozygous familial hypercholesterolemia is also a disease with varied clinical presentations, and even with appropriate treatment, it has been reported to present with an accelerated course of AV calcification and stenosis^49^. In children, a case of tuberous xanthoma initially presenting as CAVD has been noted, highlighting that the disease is not limited to the elderly^50^. Moreover, it is well documented that alkaptonuria presents with AV calcification, a condition popularly referred to as cardiac ochronosis^51^. Rare hereditary syndromes such as Singleton Merten syndrome must be considered when investigating familial cases of AS, as they present with difficult-to-group clinical features^52^. Aside from hereditary conditions, early-onset chronic renal disease with impaired calcium metabolism has been shown to present with rapidly progressive AV stenosis^53^.

A case of transient ischemic attack secondary to CAVD, possibly secondary to calcific emboli affecting the cerebral arteries, has been reported^54^. Heyde syndrome, a well-known cardiac manifestation of CAVD, has been reported to present with iron deficiency anemia, possibly due to chronic gastrointestinal blood loss from the angiodysplasias that develop due to the disease^55^. A case of recurrent ventricular arrhythmias has been noted in association with a calcified AV, which demonstrated resolution after AV reimplantation, indicating that such arrhythmias could be sequelae of AV calcifications^56^. A similar case of syncope due to paroxysmal AV block was also noted to be associated with calcified AV. An interesting case was reported in which a patient with CAVD presented with infertility. Upon further investigation, the cause was demonstrated to be calcifications of the bilateral vas deferens, thus highlighting a possible link between cardiac diseases and infertility^57^.

### Non-cardiac features

In addition to its usual cardiac features, AS can have systemic manifestations. One of the most well-described non-cardiac manifestations of AS is that of Heyde syndrome, which presents as a triad of AS, angiodysplasias with associated bleeding, and acquired von Willebrand syndrome^58,59^. Iron deficiency anemia can also be observed in patients with AS, either secondary to long-standing gastrointestinal bleeds or due to other lesser-understood mechanisms^60^. AS has also been associated with the development of cognitive impairment in rigorous clinical testing, which improves with disease resolution. Moreover, CAVD can present as embolic stroke secondary to calcified embolus derived from AV calcifications^61,62^.

### Clinical examination

As most cases of AS are asymptomatic until late in the disease course, a detailed clinical examination provides great help in screening for the disease. The findings of clinical examinations can be categorized as follows:

#### Palpation

Palpatory findings are more prominent later in the disease course. Structural changes in the heart result in a laterally displaced apical impulse due to LV hypertrophy, and in some cases, a double apical impulse may also be noted. Pulsus parvus et tardus, or a slow-rising carotid pulse with a delayed peak, may be palpated as the disease progresses and is one of the pathognomonic signs of the disease^63^. A case of visible pulsus parvus and tardus has also been reported^64^.

#### Auscultation

Although lacking specificity or sensitivity, auscultatory findings are one of the first signs noticed whenever AS is suspected. In fact, a comparative study demonstrated that auscultation is comparable, if not superior, in identifying stenotic valvular lesions^65^. The classic murmur of AS is a systolic, crescendo-decrescendo murmur often heard best in the second right intercostal space, which radiates to the carotid arteries^63^. A murmur with musical quality heard prominently at the apex (Gallavardin phenomenon), which may be confused with the murmur of mitral regurgitation (MR), may accompany the basal murmur in a minority of cases. This murmur must be differentiated from MR, which has a harsh holosystolic characteristic. Paradoxical splitting of the second heart sound may also be achieved with appropriate maneuvers^63^. In patients with BAV, an early systolic ejection murmur may be heard prior to the development of AS^66^.

### Complications of CAVD

CAVD is associated with increased cardiovascular mortality and morbidity. Untreated disease can lead to significant complications ranging from sudden cardiac death to infective endocarditis. Sudden cardiac death may occur, even in asymptomatic individuals with no classic clinical features of the disease^8^. Calcification of the valve and the resulting stress lead to an increased predilection for the development of infective endocarditis in these patients.

Even after catheter-based valve replacement, the rate of heart failure development was higher in patients with CAVD. Myocardial ischemia and myocardial infarction are also observed in untreated patients and have an overall worse prognosis than those without coexisting AS^67^. Emboli from calcified AV can lead to the development of embolic stroke in these patients.

### Investigations and imaging

Patients with AS typically present with a triad of symptoms, including angina pectoris, syncope, and dyspnea^68^. The presence of left-predominant features may indicate heart failure. Murmurs similar to those observed in AS may be heard in patients with pulmonic stenosis and hypertrophic obstructive cardiomyopathy; therefore, multiple clinical maneuvers are commenced for investigation and appropriate management^69^. Auscultation during inspiration increases murmurs in the case of pulmonary valve stenosis; innocent murmurs, on the contrary, would be decreased after standing in the supine position for a minute^70^, showing that innocent murmurs were found in approximately 65–85% of young school-aged children^70,71^. The Valsalva maneuver, forcing expiration against a closed glottis, may be used because it reduces preload and end-diastolic volume, which aids in differentiating between AS and hypertrophic obstructive cardiomyopathy, since murmurs are accentuated in response to the Valsalva maneuver in the latter, due to reduced end-diastolic volume^72^.

### Chest X-ray

The initial diagnosis of AS is usually confirmed by chest radiography (CXR), especially in symptomatic undiagnosed patients. However, radiographic findings may not be significant in the initial stages due to a lack of compensatory mechanisms, yet they are typically found in cases of advancement and when heart failure occurs. Therefore, since CXR is a nonspecific tool, it is not recommended as a sufficient diagnostic tool for AS, despite its role in the initial diagnosis^73^.

### Electrocardiography

A study by Cohen-Shelly *et al*.^74^ used electrocardiography (ECG) with artificial intelligence (AI) for AS screening and successfully identified patients with moderate to severe AS. ECG is found to be an essential tool in detecting comorbidities such as coronary heart disease, yet it is not deemed a sufficient diagnostic tool for AS^72^. LV hypertrophy may be indicated, yet not reliably, through ECG by the Sokolow-Lyon voltage positive finding (Sv1 + RV5 or 6 >3.5 mV) and strain pattern, which were both found to be associated with AS, according to a study conducted by Mino *et al*.^75^.

### Echocardiography

Echocardiography remains a tool for definitive and confirmed diagnosis of AS^42,44^. Echocardiographic imaging provides a reliable evaluation of valve anatomy, motion, and the extent of obstruction and is hence considered the gold standard for AS diagnosis. Nevertheless, AS severity could be underestimated or, contrastingly, less often overestimated in hypertensive patients; therefore, imaging must be conducted under controlled blood pressure conditions to avoid the influence of confounding flow effects^42,44^. Clinical decision-making pertaining to AS patients is as follows: (1) maximum aortic velocity, (2) mean pressure gradient (Bernoulli equation is employed for calculation), (3) valve area (continuity equation is employed for calculation), (4) ratio of velocity in the LV outflow tract proximal to the AV, and (5) velocity in a narrowed aortic orifice, when discrepancies in the aforementioned measurements or in other clinical/imaging data are encountered^42,76^.

The American College of Cardiology (ACC)/American Heart Association (AHA) 2020 guidelines recommend the following routine for patients with asymptomatic AS and normal LV function^42^ (Table 1).

**Table 1:** Frequency of ECG in asymptomatic AS patients with normal LV function

Stage^a^	Time interval for ECGs
Progressive (Stage B)	• Every 3–5 years for mild severity; *V*_max_ is 2.0–2.0 m/s • Every 1–2 years for moderate severity; *V*_max_ is 3.0–3.9 m/s
Severe asymptomatic (Stage C1)	• Every 6–12 months; *V*_max_ ≥4 m/s

a Patients with mixed valvular disease may require multiple evaluations at earlier intervals. The guideline intervals above do not consider the etiology of valve disease.

### CT scan

Cardiac computed tomography (CCT) imaging provides insights into the diagnosis of AS and provides deeper details of pre-interventional planning^73^. Valvular calcification scored through CCT could further aid in assessing the severity of the patient’s case, since it is found to be a predictor for the progression and prognosis of AS. Moreover, non-contrast calcium scoring of the aortic valve (CT-AVC) is recommended to evaluate AS severity, especially when echocardiography measurements are discordant^77^. In up to 40% of cases, patients have discordant echocardiographic assessments of AS, therefore warranting clinical uncertainty, highlighting the role CT-AVC plays, especially because of its imperative advantage of being independent of hemodynamic status. Nevertheless, it is crucial to acknowledge that the first-line and gold standard assessment of AS is Doppler echocardiography^78^. Meanwhile, contrast CT is considered the gold standard anatomical assessment prior to transcatheter AV intervention^78,79^.

### Magnetic resonance imaging scan

Cardiac magnetic resonance (CMR) imaging is a useful, non-irradiating, non-invasive modality to assess the severity of AS through two parameters: (1) planimetry of the valve area and (2) peak velocity across the AV, the first being a faster procedure^80^. Therefore, CMR is considered an effective alternative to invasive techniques, such as transesophageal echocardiography and cardiac catheterization, in the context of AS, although echocardiography remains the cornerstone for AS evaluation^81^. Different CMR modalities are compared in Table 2, adapted from the American Heart Association^81^.

**Table 2:** CMR modalities’ advantages and limitations

Technique utilized	Advantages	Limitations
Aortic valve planimetry	• Reproducible • Useful in discordant echocardiographic findings or poor acoustic windows • Provides direct visualization of the stenotic orifice	• Arrhythmia • Nonplanar orifice and different from effective orifice area • Highly calcified valves
Left ventricle (LV) remodeling	• Reproducible • Considered as gold standard for wall thickness and LV mass • Useful in misaligned LV, asymmetrical hypertrophy, and poor acoustic windows	• Time consuming • Claustrophobia • Less available • High cost • Respiratory artifacts
Late gadolinium enhancement	• Non-invasive technique for LV fibrosis quantification and visualization • Prognostic information	• Nephrogenic systemic fibrosis may occur in severe renal failure • Gadolinium injection is needed and therefore intravenous access • Focal fibrosis is only evaluated
Phase-contrast	• Reproducible • Useful in discordant echocardiography findings or poor acoustic windows • Concordant with transthoracic echocardiography	• Time consuming • Specific sequences • Incorrect placement may lead to underestimating velocity • High flow velocities may lead to underestimating AS severity • Eccentric blood flow jets
T1 mapping	• Reproducible • Interstitial fibrosis and extracellular volume calculation (ECV) • Helpful in assessing progression of disease and response to aortic valve replacement	• Specific sequences • Various protocols available • Local reference must be established for T1 mapping values with healthy subjects • Recent hematocrit value must be provided for ECV calculation
4-Dimensional (4D) flow	• Quantified blood dynamically, with full access to three-directional blood flow velocities • Eccentric blood flow jets are assessed • Flow dynamics and repercussion on aortic wall are better understood	• Time consuming • Specific sequences • Low availability • Software needed for sequence and analysis, and there is a variety • Operator dependency

Although endomyocardial biopsy is considered the gold standard for assessing myocardial fibrosis, a hallmark of severe AS with an important prognostic role, the presence of focal or diffuse fibrosis could be non-invasively evaluated using CMR^81^.

### Definitive diagnosis of CAVD

The diagnosis of CAVD is primarily based on echocardiographic examinations, which aid in visualizing the valve anatomy and severity of valve calcification. Longitudinal systolic strain imaging is a more sensitive indicator of LV function and is effective in anticipating unfavorable outcomes such as mortality^82^. Doppler echocardiography serves as the primary modality for assessing AS severity. However, in cases where the echocardiography results are inconclusive or conflicting, cardiac catheterization may be employed to confirm the hemodynamic severity of the stenosis. Cardiac catheterization facilitates the measurement of cardiac blood pressure and flow, aiding in precise diagnosis^42^.

CMR imaging is valuable in scenarios where echocardiographic data are suboptimal or contradictory to the clinical evaluation of AS severity. CMR is effective in distinguishing between bicuspid and trileaflet valve anatomies while also providing an accurate assessment of peak jet velocity. Furthermore, CMR is beneficial for evaluating the anatomy of the aortic root and ascending aorta, particularly in patients with a bicuspid valve^83^.

CT imaging offers an alternative approach for assessing valve area through planimetry and provides quantitative measurements of valve calcification. For patients undergoing transcatheter aortic valve replacement (TAVR), a multimodal imaging approach includes CT assessment of the aortic annulus size and shape, leaflet length, and the distance between the annulus and coronary ostia.

The standard criteria used to assess the severity of AS include the maximum velocity (*V*_max_) across the stenotic valve, the mean transaortic pressure gradient (Δ*P*_mean_) calculated using the Bernoulli equation, and the functional aortic valve area (AVA) determined using the continuity equation. Notably, echocardiographic calculations of Δ*P*_mean_ and AVA have been rigorously validated against invasive measurements and are now considered the established clinical practice standards^42^.

The international clinical guidelines endorse the evaluation of AS severity using both echocardiography and CT-AVC scoring when echocardiographic assessments yield discordant results. CT-AVC has emerged as an alternative means of assessing AS severity, offering a structural assessment that is independent of flow, focusing on the quantification of the calcium burden within the valve. When employing sex-specific thresholds, CT-AVC demonstrates good accuracy compared with echocardiography and provides significant prognostic information. Additionally, 18F-sodium fluoride uptake in the AV, as observed through positron emission tomography (PET), aids in identifying active tissue calcification. Moreover, it predicts changes in AV calcification on follow-up CT scans conducted 1–2 years later.

### Treatment

The management spectrum of CAVD is evolving constantly. Treatment modalities range from surgical interventions and medical management to palliative and transient relief services, such as aortic balloon valvuloplasty^84,85^. Simvastatin and ezetimibe combination therapy reduces ischemic events in mild-to-moderate AS but not in severe cases^12^. Elevated Lp(a) levels are known to potentiate the atherosclerotic process of CAVD. Pharmacological interventions targeting Lp(a), such as proprotein convertase subtilisin/kexin type 9 (PCSK9) inhibitors, slow the progression of CAVD and reduce the dependence on surgical treatment. However, interventions remain the gold standard of treatment for symptomatic patients with CAVD^84,85^.

Although the role of valvuloplasty has significantly reduced considering the development of TAVR, it is known to cause a modest increase in the AV area and reduce transvalvular pressure, providing short-term relief to patients. It neither improves the mortality rate nor offers any survival advantage. Aortic balloon dilatation is performed in children to avoid disrupting growth before definitive surgical valve replacement^86^.

The indications for AVR include symptomatic patients, asymptomatic patients with severe AS, patients undergoing cardiac surgery for any other cause, classic and low-flow low-gradient AS, or decreased blood flow velocity across the AV by at least 0.3 m/s per year^86^.

Artificial valves can be bioprosthetic or mechanical. The choice of valve depends on the patient’s age and well-being. Mechanical valves, such as balls and cages and bi-leaflets, are more durable but can predispose to thrombosis and embolism; thus, there is a need for lifelong anticoagulant therapy. Bioprosthetic valves employed in AVR could either be balloon-expanding, such as Sapien, or self-expanding, such as CoreValve^87^. They could either be allografts or xenografts. The Ross procedure is generally employed for congenital AS, where the diseased AV is replaced with the patient’s own pulmonary valve (autograft) and the pulmonary valve is replaced with an aortic or pulmonary allograft. The most commonly used xenografts were bovine and porcine pericardial valves^88^.

The major advantage of bioprosthetic valves is their ability to improve hemodynamics and blood flow considerably, postoperatively with no lifelong need for anticoagulants. However, they are less durable and prone to calcification on the valve leaflets^88^.

Due to the current technological advancements, more durable, less thrombogenic, and better hemodynamic stability-oriented valves are being researched. Obtained from the skin graft of burn victims, tissue-engineered heart valves are also infection-resistant. Culturing techniques may even reveal the tendency of these valves to repair themselves^88^.

AVR can be performed either via open-heart surgery or catheter insertion. Surgical AVR (SAVR) involves cardiopulmonary bypass (CPB) and cardioplegic arrest. Performed under general anesthesia, SAVR is the conventional treatment for patients with low or intermediate surgical risk. The damaged AV was replaced with either a mechanical or a biological artificial valve. Owing to the risks involved in performing open-heart surgery and associated complications postoperatively, a minimally invasive technique was sought after, which led to the development of TAVR^87^.

TAVR is performed percutaneously under general or local anesthesia depending on the route of insertion. The most common route is the transfemoral approach, which is performed under general anesthesia. TAVR involves the placement of a collapsible bioprosthetic AV inside the valve. During the expansion of the new valve, the narrowed valve was pushed outward, taking charge of the blood flow from the LV to the aorta^87^.

Other routes, such as transthoracic or transapical, can be employed when the femoral artery cannot be used due to any abnormalities in the vasculature factors, such as size, tortuosity, or possible chance of calcification. TAVR was originally developed for patients at high surgical risk, such as those with advanced age, comorbidities, or patient rejection. It is now widely accepted by patients across the entire spectrum of severity owing to better clinical outcomes and minimally invasive interventions^87^. Guideline recommendations should aid in the treatment of associated cardiac conditions such as heart failure, atrial fibrillation, and hypertension. In cases of resistant hypertension with concomitant atrial fibrillation or heart failure, renal denervation might be a suitable modality choice to prevent the worsening of symptoms of AS and acute decompensation due to the superimposition of multiple pathologies in a single patient^89^.

## Emerging therapeutic modalities for calcific AS

### Therapies

The therapies are summarized in Table 3. Ataciguat (HMR1766) is a guanylyl cyclase activator^90^ used in the management of pulmonary hypertension^91^ because of its relaxant effects. Ataciguat may be useful in reducing blood pressure in patients with moderate CAVD (Clinicaltrials.gov ID: NCT02049203). An ongoing clinical trial (Clinicaltrials.gov ID: NCT04913870) evaluated the efficacy of angiotensin receptor blockers (ARBs) on mild-to-moderate AS and to slow AS progression and LV remodeling. Antifibrotic therapy (spironolactone and dihydralazine) is being evaluated in a phase-3 trial, Reduce-MFA, to assess their effect on the regression of myocardial fibrosis after TAVI using ECV-derived matrix volume (measured by CMR) (Clinicaltrials.gov ID: NCT05230901). Eplerenone, a selective aldosterone receptor antagonist, has been studied as a potential therapeutic agent in patients with impaired cardiac hemodynamics after TAVR (Clinicaltrials.gov ID: NCT03923530).

**Table 3:** Ongoing drug trials for calcific aortic valve disease

Study contact and institution	Trial ID	Year	Drug	Study description/status
Jordan D. Miller, Mayo Clinic	NCT02049203	2016	Ataciguat (HMR1766)	Ataciguat guanylyl cyclase activator and may be useful in reducing blood pressure in patients with moderate calcific aortic valve stenosis (CAVS)
Marie-Annick Clavel, Institut universitaire de cardiologie et de pneumologie de Québec, University Laval	NCT04913870	2022	Angiotensin receptor blockers (ARBs)	An ongoing phase-4 trial evaluating the efficacy of ARBs on mild-to-moderate AS and to slow AS progression and left ventricular remodeling
Karsten Gavenis, University Medical Center Goettingen	NCT05230901	2022	Spironolactone and dihydralazine	An ongoing phase-3 trial, evaluating the ability of combination therapy (spironolactone and dihydralazine) to reduce-MFA, and to assess their effect on the regression of myocardial fibrosis after TAVI using ECV-derived matrix volume (measured by CMR)
Anthony Bavry, North Florida Foundation for Research and Education	NCT03923530	2020	Eplerenone	A completed trial, studying eplerenone, a selective aldosterone receptor antagonist, as a potential therapeutic agent in patients with impaired cardiac hemodynamics after TAVR
Zhi Jian Wang, Beijing Anzhen Hospital	NCT04968509	2024	PCSK9 inhibitors	EPISODE trial, in its 3rd phase, assesses the effect of PCSK9 inhibitors on calcific aortic valve disease based on the established reduction in atherogenic lipoprotein cholesterol-carrying particles such as lipoprotein(a)
Novartis (Novartis Pharmaceuticals)	NCT05646381	2023	Pelacarsen (TQJ230)	An ongoing trial to assess the impact of reducing lipoprotein(a) in patients with CAVS and to measure the change in aortic valve calcium score. The study also aims to evaluate efficacy, safety, and tolerability of pelacarsen (TQJ230)
Ottawa Heart Institute Research Corporation	NCT05253794	2023	Colchicine	A pilot single-center double-blinded randomized study to determine the effect of targeted anti-inflammation therapy using colchicine, on valvular calcification activity using imaging
Fei Li, Wuhan Union Hospital, China	NCT05875675	2023	Pioglitazone	A phase-2 trial evaluating pioglitazone’s ability to slow the progression of aortic valve calcification because pioglitazone has anti-inflammatory and antioxidant effects that may prevent cardiovascular diseases
Jae-Kwan Song, Asan Medical Center	NCT04521452	2020	Evogliptin	A phase-4 trial to assess the progression of AV calcification in patients with T2DM with mild-to-moderate CAS
Fei Li, Wuhan Union Hospital, China	NCT05861648	2023	Folic acid	Evaluating the role folic acid plays in slowing the progression of AV calcification
Konstantinos Toutouzas, National and Kapodistrian University of Athens	NCT04429035	2021	Vitamin K2 (menaquinone)	Evaluating the role vitamin K2 (menaquinone) plays in slowing the progression of AV calcification
Nicolas M Van Mieghem, Erasmusm Medical Center	NCT04171726	2019	Edoxaban	A phase-3 ongoing trial investigating the efficacy of edoxaban in reducing the incidence of leaflet thickening

EPISODE trial, in its 3rd phase, assesses the effect of PCSK9 inhibitors on CAVD (Clinicaltrials.gov ID: NCT04968509) based on the established reduction in atherogenic lipoprotein cholesterol-carrying particles such as Lp(a)^92^ with the use of PCSK9 inhibitors. The results of this ongoing trial might necessitate potential therapeutic alterations to the current guidelines for AS management. Another lipid-lowering medication, pelacarsen (TQJ230), is being used to assess the impact of reducing Lp(a) in patients with CAVD and to measure the change in AV calcium score (Clinicaltrials.gov ID: NCT05646381).

Although Pawade *et al*.^93^ concluded that denosumab and alendronic acid did not affect the progression of AV calcification in patients with CAVD, their trial was based on studies showing that bone turnover and osteoblastic differentiation of VICs are significant contributors to the pathogenesis of CAVD. Li *et al*.^94^ reported significant reductions in serum C-terminal telopeptide, a measure of bone turnover, with the use of denosumab and alendronic acid, but no significant changes were observed in the AV calcium score. Potentially larger and longer multicenter trials might conclude some efficacy of osteoporosis drugs in slowing AS progression.

COPAS-Pilot is an ongoing trial assessing the effect of colchicine on valvular calcification activity using aortic valvular NaF uptake (Clinicaltrials.gov ID: NCT05253794). CAVD mouse models have established the role of targeted PTPN22 inhibition in reducing the progression of CAVD and, therefore, might be a potential therapeutic agent in humans with CAVD^95^. Myeloid cell reprogramming in AS is another area of interest (NCT04717219), which currently focuses on the role of activation of the innate immune system in CAVD. The outcomes of this trial will provide potential immune checkpoints, the inhibition or modulation of which might guide future CAVD research in developing these targeted therapies.

Pioglitazone, an antidiabetic medication, is being evaluated in a phase-2 trial (Clinicaltrials.gov ID: NCT05875675) to slow the progression of AV calcification because pioglitazone has anti-inflammatory and antioxidant effects that may prevent cardiovascular diseases. Another antidiabetic medication, evogliptin, is in a phase-4 trial to assess the progression of AV calcification in patients with T2DM with mild-to-moderate CAVD (Clinicaltrials.gov ID: NCT04521452). Similarly, folic acid (Clinicaltrials.gov ID: NCT05861648) and vitamin K2 (menaquinone) (Clinicaltrials.gov ID: NCT04429035) have been evaluated in clinical trials to slow the progression of AV calcification. One phase-3 ongoing trial, REDOX-TAVI (Clinicaltrials.gov ID: NCT04171726), is investigating the efficacy of edoxaban in reducing the incidence of leaflet thickening. The results of REDOX-TAVI will provide therapeutic insights into the anticoagulant agents for TAVI.

Upcoming AV system includes TaurusOne transcatheter aortic valve system (Clinicaltrials.gov ID: NCT04815785), Edwards SAPIEN 3 ultra transcatheter heart valve (THV) (Clinicaltrials.gov ID: NCT03003299), Microport CardioFlow VitaFlow II Transcatheter Aortic Valve System (Clinicaltrials.gov ID: NCT04414878), ACURATE neo2 transfemoral TAVR system (Clinicaltrials.gov ID: NCT03735667), Microport CardioFlow VitaFlow II Transcatheter Aortic Valve System (Clinicaltrials.gov ID: NCT04414878), GEMINUS transcatheter aortic valve implantation system (Clinicaltrials.gov ID: NCT05909748), Myval transcatheter heart valve system (Clinicaltrials.gov ID: NCT04703699), Medtronic Evolut PRO+ TAVR system (Clinicaltrials.gov ID: NCT05149755), Vienna aortic valve SE system (Clinicaltrials.gov ID: NCT04861805), Colibri TAVI system (Clinicaltrials.gov ID: NCT04029844), INOVARE transcatheter valve (Clinicaltrials.gov ID: NCT05531578), Navitor transcatheter aortic valve (Clinicaltrials.gov ID: NCT04788888), and ALLEGRA THV system (Clinicaltrials.gov ID: NCT05478161).

To reduce cerebral embolic complications in TAVR, an ongoing phase-1 trial is evaluating the safety and efficacy of the F2 device, which is positioned in the aorta and covers the three great cerebral vessels during TAVR (Clinicaltrials.gov ID: NCT05866640). Other similar cerebroprotective devices being tested are AorticLab FLOWer system (Clinicaltrials.gov ID: NCT04704258) and EMBLOK EPS (Clinicaltrials.gov ID: NCT05295628). Another trial assessed the outcomes of transcatheter AV flushing with CO_2_ (TAVI-CO_2_) versus standard saline flushing of valves (TAVI-S), as CO_2_ use in cardiac interventions has shown beneficial effects (Clinicaltrials.gov ID: NCT05146037).

To reduce paravalvular regurgitation (PVR) after TAVR, the WITAVI-REAL clinical trial (Clinicaltrials.gov ID: NCT03728049) evaluated the efficacy of point-of-care testing of von Willebrand factor as an alternative to a transesophageal echocardiogram at the time of minimally invasive TAVR in reducing PVR post-TAVR. Porcine calcified-aortic models have been demonstrated to reverse elastin-specific medial vascular calcification (Monckeberg’s sclerosis) by local periadventitial delivery of chelating agents, such as disodium ethylenediaminetetraacetic acid (EDTA), loaded into poly(lactic-co-glycolic acid) nanoparticles^94^. Successful reversal of calcification in human aortic tissue may have massive clinical implications. Future research should focus on developing therapeutics that can reverse calcification, which will lead to major progress in the management and improvement of the prognosis of CAVD. Further research needs to be conducted on upcoming therapies in cardiovascular medicine, such as bempedoic acid and PCSK9 inhibitors like inclisiran, to determine their potential role in managing CAVD^96^.

### Modalities

The incorporation of digital technology into AS is a recent trend. An ongoing clinical trial (Clinicaltrials.gov ID: NCT05230225) has assessed the impact of electronic physician notification letters on the likelihood of AVR utilization in patients with a clinical indication for severe AS. Novel CAVD biomarker kits are being prepared to assess the severity and prognosis of AV stenosis (Clinicaltrials.gov ID: NCT04312139) using multiplexed enzyme-linked immunosorbent assay (ELISA) kits. Novel non-invasive ultrasound therapy (NIUT) with Valvosoft is being evaluated for safety in patients with severe CAVD (Clinicaltrials.gov ID: NCT05235568). The outcomes of this trial assessing NIUT with Valvosoft could potentially provide data that reduce the necessity of TAVR in certain severe CAVD cases.

The ART-VR trial is an ongoing trial that estimates the effect of an immersive virtual reality (VR) environment on reducing procedural anxiety in patients undergoing TAVR (Clinicaltrials.gov ID: NCT05069987). With advances in AI, computerized stethoscopes (©VoqX) are undergoing clinical trials to diagnose structural cardiac pathologies (Clinicaltrials.gov ID: NCT04960280). ©VoqX is being compared with the current gold standard testing, such as echocardiography or invasive cardiac catheterization. An ongoing trial tested the HUAWEI smartwatch to facilitate early discharge in patients following TAVR by assessing conduction disturbances, daily activity levels, heart rates, oxygen saturation, and sleep patterns (Clinicaltrials.gov ID: NCT04454177).

PET imaging with 18F-NaF has been demonstrated to quantify disease activity; since disease activity is a predictive marker of future disease progression, PET-CT has now become crucial for prognostication of CAVD^97^. Doris *et al*.^98^ optimized 18F-NaF PET by reconstructing parameters and cardiac motion correction and demonstrated improvements in signal-to-noise ratio, which consecutively led to improvements in blurring from cardiac motion^98^.

The search for novel radiotracers that target inflammation, thrombus formation, and fibrosis is crucial for reducing the complications of CAVD. 18F-GP1 is a novel tracer for PET imaging that binds to glycoprotein IIb/IIIa receptors on activated platelets during thrombotic depositions^99^. Another upcoming tracer is 68Ga-DOTATATE, which targets the somatostatin receptor expressed on activated macrophages during the pathogenesis of atherosclerotic inflammation^97,100^. 68Ga-DOTATATE-PET has been demonstrated to have superior coronary imaging efficacy than 18F-FDG-PET, with better power in discriminating high-risk coronary plaques.

### Progression, prognostication, and survival characteristics

CAVD is divided into two separate stages. The first stage involves lipid accumulation and endothelial failure, and the second stage involves calcification, fibrosis, and inflammation^15^. Endothelial failure in valvular endothelial cells is the first sign of calcification and is caused by several risk factors, including mechanical/shear stress, lipid accumulation, ROS, and inflammation. The progression phase, which follows the initial inflammatory phase, begins with the conversion of VICs into myofibroblastic and osteoblastic phenotypes, a process governed by the cytokines produced by immune cells^11^.

Women display less severe inflammatory and fibrotic responses than men when it comes to cardiac remodeling. When triggered by interferon-α, female VICs show a tendency to be less prone to calcification. This distinction is related to sex-specific apoptotic pathways such as the PI3K/Akt survival pathway^100^. Lower levels of calcification in female AVs may be caused by variations in the secretion of important factors, such as IL-6, BMP-2, and MMP-1. Interferon-gamma (IFN)-induced pro-angiogenic, pro-inflammatory, and pro-calcific actions have a greater impact on male VICs. Women also exhibit reduced profibrotic TGF expression and a milder inflammatory response^17^.

As such, the balance between inhibitors and promoters is critical for calcification development owing to near-supersaturated calcium and phosphate concentrations. A primary systemic inhibitor, fetuin-A, prevents calcification by generating soluble protein–mineral nanoparticles. Low fetuin-A levels reduce calcification inhibition, leading to calcification when excessive. Koos *et al*.^101,102^ linked fetuin-A serum levels with AV calcification progression and clinical outcomes independent of renal function and inflammation. Fetuin-A levels can be utilized as a useful parameter with defined levels for clinical protocols, because higher levels are associated with better survival.

Numerous prospective studies have been conducted to increase the predictability of disease progression, particularly in AS. However, because each of these studies had a substantial margin of error, it was difficult to predict the progression of AS. Pasch *et al*.^103^ developed a novel in-vitro blood test, the T50 test, which assesses calcification propensity by monitoring the maturation time of calcium particles. Particularly, in patients with aortic sclerosis, this test is promising as a biomarker for predicting future arterial calcifications (Clinicaltrials.gov ID: NCT02241109). Another approach, known as PET-MDCT, combines anatomical and molecular imaging to evaluate the uptake of 18F-sodium fluoride by valvular tissue, which reveals the presence of active mineralization processes within the valve. This predicts the progression of AS and correlates with the severity of the condition^16^. Emerging blood biomarkers, including high-sensitivity cardiac troponin, growth/differentiation factor 15, ST2 protein, and miRNAs, also have the potential to identify myocardial dysfunction, although further research is required to determine their relevance beyond that of already established indicators^11^. It is critical to keep in mind that these markers are not very specific and can be affected by other health issues such as hypertension, DM, and coronary artery disease. Despite their shortcomings, these techniques present new opportunities to improve the prognosis of AS progression and associated cardiac events.

Regarding AS diagnosis and classification, valve anatomy, hemodynamic severity, LV response, and symptoms were considered. AS can range in severity from mild to severe with or without symptoms^104^. The primary method for determining AS severity is Doppler echocardiography. If the results are unclear, cardiac catheterization, which measures heart blood flow and pressure, may be employed, although this is hazardous. Peak aortic jet velocity, mean gradient, and AVA are the three variables used by Doppler to gauge the severity of AS. As a result of the faster flow (higher peak velocity) and pressure loss (increased mean gradient) caused by the calcific AS, the AVA gradually decreased. An increased peak velocity/mean gradient along with thicker AV leaflets with constrained opening are diagnostic indicators of AS. Echocardiography aids in determining how AS affects the LV shape and functionality. Patients with mild-to-moderate AS typically do not have any symptoms unless other conditions are present. They had a peak velocity of less than 4 m/s, a mean gradient between 25 and 40 mmHg, and an AVA between 1 and 1.5 cm^2^. Patients with severe AS, commonly defined as a peak velocity of 4 m/s, mean gradient of 40 mmHg, and an AVA of 1 cm^2^, may or may not have symptoms and require more frequent clinical and Doppler monitoring. Doppler echocardiography is a useful method for assessing AS severity because it can calculate the maximal instantaneous and mean AV gradients using continuous-wave Doppler velocity across the valve^104^.

### Quality of life

With the increasing prevalence of the disease and significant mortality rates, CAVD caused 1.5 million (95% uncertainty interval 1.4 million–1.6 million) DALYs globally in the year 2017. The percentage change in DALYs in 2017 compared with 1990 was 101%^104^.

Heart failure is the most common complication associated with severe AS, causing significant morbidity and commonly presenting with fatigue, shortness of breath, orthopnea, and pedal edema as valvular disease progresses. This significantly impairs quality of life (QoL) by decreasing the overall functional capacity, especially in elderly patients^105^. The overall QoL may vary in the elderly due to associated morbidities, such as DM, obesity, and renal diseases, which by themselves are risk factors for CAVD, as previously mentioned^106^, or due to frailty^107^. It also contributes to the surgical risk in patients requiring AVR^108^. The psychosocial impact of this disease should not be overlooked. Patients can develop anxiety and depression owing to limited physical activity and the risk of sudden cardiac events^108^.

With the availability of AVR, there have been substantial improvements in QoL and survival rates in patients with severe aortic valvular disease and heart failure. TAVR has become the treatment of choice in patients at high risk of SAVR because of its minimally invasive technique, lower complications, and better overall long-term outcomes. Some patients may experience symptoms of residual heart failure due to diastolic dysfunction with preserved ejection fraction, but they are comparatively less severe^109^.

Several considerations must be made while determining the potential benefits of AVR, including pre-procedure symptom severity due to valvular disease and health status. Identifying the lesion and considering AVR early before the development of irreversible cardiac changes improves the prognosis and QoL^110^. AVR increases long-term risks for subclinical leaflet thrombosis^111^ and infective endocarditis^112^, leading to embolism, including stroke, and further increasing morbidity. Based on these indications, the use of oral anticoagulants for thrombosis^111^ and the case-by-case use of antibiotic prophylaxis^112^ help prevent potentially life-threatening complications. Comprehensive care, support networks, and social interventions should address the psychosocial aspects to improve QoL. Exercise training-based cardiac rehabilitation is known to improve functional capacity in frail patients who have undergone AVR, thus improving their QoL^109^. Theoretically, high-dose eicosapentaenoic acid slows disease progression^106^, but its clinical benefits need to be studied. Further research is required to identify the factors affecting pre-AVR and post-AVR QoL and to explore other therapies, particularly those focusing on non-cardiac variables.

### Challenges in the myriad of CAVD

Innovative diagnostic approaches have emerged in response to the challenges posed by CAVD and coexisting heart valve issues. These methods incorporate advanced technologies and target CAVD-specific biomarkers to enhance the effectiveness of TAVR treatment. Traditional echocardiography, designed for single-valve issues, can be misleading when multiple valve diseases coexist with CAVD. The precise assessment of conditions such as MR, mitral stenosis (MS), and aortic regurgitation (AR) is crucial. Stress echocardiography aids in distinguishing genuinely severe AS from potentially less severe cases. CCT calculates AV calcium scores, while CMR assesses regurgitant lesions, and heart chamber changes, and detects replacement fibrosis through late gadolinium enhancement (LGE) and ECV measurement, offering crucial prognosis insights. A complete imaging approach is crucial for precise diagnosis and treatment guidance in patients with AS^113^. TAVR patients in the United States are now younger and healthier, benefiting from improved short-term outcomes due to better devices and procedures. Patient characteristics play a larger role in 1-year outcomes. TAVR programs have expanded due to volume requirements and procedural excellence, with experience showing a learning curve. Comprehensive metrics beyond 30-day mortality are essential for assessing quality of care. Collaboration and best practices should strengthen low-volume TAVR programs, considering patient-centered outcomes such as QoL. Technological advancements have resulted in improved TAVR results^114^.

Six key biomarkers, Irisin, Periostin, Osteoglycin, IL-18, HMGB1, and PCSK9, shed light on intricate mechanisms and offer diagnostic and prognostic insights. Specific serum- and valve-specific biomarkers, such as IL-6Rα, uPAR, ET-1, and Gal-3, provide valuable information for assessing disease severity and predicting outcomes. Gender-specific patterns have been identified, with GDF-15, sST2, and NT-proBNP offering prognostic value for AS^114–118^.

BNP and troponin levels aid in assessing heart failure severity and maladaptive remodeling in patients with AS. Targeted therapies include lipid-lowering strategies, RAAS inhibitors, and metabolic agents. NO-cGMP signaling modifiers, interventions targeting the NOTCH pathway, and non-coding RNAs provide additional avenues for therapeutic development. Efforts to manage mineral metabolism and break the calcification cycle are underway, with bisphosphonate and vitamin K2 supplementation being explored. Controlling hypertension and heart rate may play a role in slowing CAVD progression^85,116,119^.

Studies including machine learning algorithms to identify novel biomarkers, investigations into the role of MMP9 in mitochondrial dysfunction, DUSP26 inhibition as a therapeutic strategy, apoC-III as a contributor to valvular calcification, and the potential role of copeptin as a risk stratifier offer further insights into CAVD management^28,120–122^.

## Conclusions

In developed countries, following coronary artery disease and systemic arterial hypertension, CAVD is the third most prevalent cardiovascular disease affecting the heart valves. DALYs associated with CAVD have continued to increase over the decades, and our understanding of their causes has evolved significantly during the 20th century. Modern world risk factors for CAVD include sedentary lifestyle, obesity, dyslipidemia, DM, and hypertension. Despite the rising prevalence of CAVD, surprisingly, most patients do not experience symptoms until the 7th or 8th decade of life. In individuals who exhibit symptoms, angina pectoris, exertional dyspnea, and syncope are hallmark features, although AS can also present in atypical ways, such as paroxysmal or recurrent arrhythmias. Although various diagnostic methods are available, echocardiography remains the definitive tool for confirming the diagnosis of AS. A comprehensive imaging approach is crucial to ensure precise diagnosis and guide treatment for individuals with AS.

The management approach for CAVD has developed continuously over time. Available treatments encompass a wide range, including surgical procedures, medical care, and palliative measures to provide temporary relief. AVR is considered to be the benchmark approach, whether through surgical or interventional procedures. As the field of cardiology continues to evolve, ongoing research has led to the investigation of several drugs showing promise in CAVD management, including PCSK9 inhibitors, ataciguat, pelacarsen, and pioglitazones. Recent advances in new TAVR systems have shown promising results in terms of mortality and complication reduction. The deployment of upcoming cerebroprotective devices has reduced the incidence of stroke post-TAVR. Extensive research is currently underway to develop reliable biomarkers that can effectively track the progression of CAVD in patients, given its association with increased mortality and complications, such as stroke. Among these biomarkers are the T50 test, high-sensitivity cardiac troponin, growth/differentiation factor 15, ST2 protein, and miRNAs. Unfortunately, these biomarkers currently lack the specificity required to be entirely reliable. With ongoing research efforts, the future of diagnostic and management options for CAVD appears promising, enabling cardiologists to offer more effective guidance to patients during challenging times.

## Ethical approval

No ethical approval was required due to the review nature of the study.

## Consent

No consent was required due to the retrospective review nature of the study based on public information.

## Sources of funding

The authors have no funding to report.

## Author contribution

H.J.: conceptualization, methodology, investigation, writing – original draft, writing – review and editing, supervision, project administration; A.G.: writing – original draft, writing – review and editing, supervision; A.T.M.A.K., N.U.K., J.J., S.C., S.A.S., K.P., K.K.: writing – original draft; M.M.R.: writing – original draft, writing – reviewing and editing; M.D.: original draft, writing – review and editing, supervision; A.H.S.: original draft, writing – review and editing, supervision, project administration.

## Conflicts of interest disclosure

The authors have no conflicts of interest to report.

## Research registration unique identifying number (UIN)

PROSPERO-CRD42023470549.

## Guarantor

Mohamed Daoud.

## Data availability statement

No data were analyzed from datasets.

## Provenance and peer review

Not commissioned, externally peer-reviewed.

## References

[1] Freeman RV Otto CM . Spectrum of calcific aortic valve disease: pathogenesis, disease progression, and treatment strategies. Circulation 2005;111:3316–3326. 15967862 10.1161/CIRCULATIONAHA.104.486738

[2] Otto CM Lind BK Kitzman DW . Association of aortic-valve sclerosis with cardiovascular mortality and morbidity in the elderly. N Engl J Med 1999;341:142–147. 10403851 10.1056/NEJM199907153410302

[3] Yi B Zeng W Lv L . Changing epidemiology of calcific aortic valve disease: 30-year trends of incidence, prevalence, and deaths across 204 countries and territories. Aging 2021;13:12710–12732. 33973531 10.18632/aging.202942 PMC8148466

[4] Yan AT Koh M Chan KK . association between cardiovascular risk factors and aortic stenosis: The CANHEART Aortic Stenosis Study. J Am Coll Cardiol 2017;69:1523–1532. 28335833 10.1016/j.jacc.2017.01.025

[5] Coffey S Cox B Williams MJA . The prevalence, incidence, progression, and risks of aortic valve sclerosis: a systematic review and meta-analysis. J Am Coll Cardiol 2014;63(25 Pt A):2852–2861. 24814496 10.1016/j.jacc.2014.04.018

[6] Ng ACT Prihadi EA Antoni ML . Left ventricular global longitudinal strain is predictive of all-cause mortality independent of aortic stenosis severity and ejection fraction. Eur Heart J Cardiovasc Imaging 2018;19:859–867. 28950306 10.1093/ehjci/jex189

[7] Faggiano P Antonini-Canterin F Ribichini F . Pulmonary artery hypertension in adult patients with symptomatic valvular aortic stenosis. Am J Cardiol 2000;85:204–208. 10955378 10.1016/s0002-9149(99)00643-8

[8] Taniguchi T Morimoto T Shiomi H . Sudden death in patients with severe aortic stenosis: observations from the CURRENT AS registry. J Am Heart Assoc 2018;7:e008397. 29776957 10.1161/JAHA.117.008397 PMC6015355

[9] Stortecky S Buellesfeld L Wenaweser P . Atrial fibrillation and aortic stenosis: impact on clinical outcomes among patients undergoing transcatheter aortic valve implantation. Circ Cardiovasc Interv 2013;6:77–84. 23386662 10.1161/CIRCINTERVENTIONS.112.000124

[10] Hudzik B Wilczek K Gasior M . Heyde syndrome: gastrointestinal bleeding and aortic stenosis. CMAJ 2016;188:135–138. 26124230 10.1503/cmaj.150194 PMC4732965

[11] Lindman BR Clavel MA Mathieu P . Calcific aortic stenosis. Nat Rev Dis Primer 2016;2:16006. 10.1038/nrdp.2016.6 PMC5127286 27188578

[12] Rossebø AB Pedersen TR Boman K . Intensive lipid lowering with simvastatin and ezetimibe in aortic stenosis. N Engl J Med 2008;359:1343–1356. 18765433 10.1056/NEJMoa0804602

[13] Lee S Lee SA Choi B . Dipeptidyl peptidase-4 inhibition to prevent progression of calcific aortic stenosis. Heart 2020;106:1824–1831. 32917732 10.1136/heartjnl-2020-317024 PMC7677484

[14] Vahanian A Beyersdorf F Praz F . 2021 ESC/EACTS Guidelines for the management of valvular heart disease. Eur Heart J 2022;43:561–632. 34453165 10.1093/eurheartj/ehab395

[15] Summerhill VI Moschetta D Orekhov AN . Sex-specific features of calcific aortic valve disease. Int J Mol Sci 2020;21:5620. 32781508 10.3390/ijms21165620 PMC7460640

[16] Sherzad AG Shinwari M Azimee MA . Risk factors for calcific aortic valve disease in Afghan population. Vasc Health Risk Manag 2022;18:643–652. 36003849 10.2147/VHRM.S376955 PMC9394646

[17] Adhikari R Shiwakoti S Ko JY . Oxidative stress in calcific aortic valve stenosis: protective role of natural antioxidants. Antioxidants (Basel) 2022;11:1169. 35740065 10.3390/antiox11061169 PMC9219756

[18] Coffey S Roberts-Thomson R Brown A . Global epidemiology of valvular heart disease. Nat Rev Cardiol 2021;18:853–864. 34172950 10.1038/s41569-021-00570-z

[19] Martinsson A Li X Zöller B . Familial aggregation of aortic valvular stenosis: a nationwide study of sibling risk. Circ Cardiovasc Genet 2017;10:e001742. 29242201 10.1161/CIRCGENETICS.117.001742 PMC5734674

[20] Peeters FECM Meex SJR Dweck MR . Calcific aortic valve stenosis: hard disease in the heart: a biomolecular approach towards diagnosis and treatment. Eur Heart J 2018;39:2618–2624. 29136138 10.1093/eurheartj/ehx653 PMC6055545

[21] Thériault S Dina C Messika-Zeitoun D . Genetic association analyses highlight IL6, ALPL, and NAV1 As 3 new susceptibility genes underlying calcific aortic valve stenosis. Circ Genom Precis Med 2019;12:e002617. 32141789 10.1161/CIRCGEN.119.002617

[22] Alushi B Curini L Christopher MR . Calcific aortic valve disease-natural history and future therapeutic strategies. Front Pharmacol 2020;11:685. 32477143 10.3389/fphar.2020.00685 PMC7237871

[23] Hulin A Hego A Lancellotti P . Advances in pathophysiology of calcific aortic valve disease propose novel molecular therapeutic targets. Front Cardiovasc Med 2018;5:21. 29594151 10.3389/fcvm.2018.00021 PMC5862098

[24] Spilias N Martyn T Denby KJ . Left ventricular systolic dysfunction in aortic stenosis: pathophysiology, diagnosis, management, and future directions. Struct Heart 2022;6:100089. 37288060 10.1016/j.shj.2022.100089 PMC10242576

[25] Aldrugh S Valle JE Parker MW . Prevalence of left ventricular hypertrophy caused by systemic hypertension preceding the development of severe aortic stenosis. Am J Cardiol 2021;150:89–94. 34052014 10.1016/j.amjcard.2021.03.036

[26] Stehli J Zaman S Stähli BE . Sex discrepancies in pathophysiology, presentation, treatment, and outcomes of severe aortic stenosis. Front Cardiovasc Med 2023;10:1256970. 37649667 10.3389/fcvm.2023.1256970 PMC10465161

[27] Raddatz MA Madhur MS Merryman WD . Adaptive immune cells in calcific aortic valve disease. Am J Physiol Heart Circ Physiol 2019;317:H141–H155. 31050556 10.1152/ajpheart.00100.2019 PMC6692729

[28] Wang Y Fang Y Lu P . NOTCH signaling in aortic valve development and calcific aortic valve disease. Front Cardiovasc Med 2021;8:682298. 34239905 10.3389/fcvm.2021.682298 PMC8259786

[29] Gollmann-Tepeköylü C Graber M Hirsch J . Toll-like receptor 3 mediates aortic stenosis through a conserved mechanism of calcification. Circulation 2023;147:1518–1533. 37013819 10.1161/CIRCULATIONAHA.122.063481 PMC10192061

[30] Blaser MC Kraler S Lüscher TF . Network-guided multiomic mapping of aortic valve calcification. Arterioscler Thromb Vasc Biol 2023;43:417–426. 36727519 10.1161/ATVBAHA.122.318334 PMC9975082

[31] Dharmarajan S Speer MY Pierce K . Role of Runx2 in calcific aortic valve disease in mouse models. Front Cardiovasc Med 2021;8:687210. 34778386 10.3389/fcvm.2021.687210 PMC8585763

[32] Phua K Chew NW Kong WK . The mechanistic pathways of oxidative stress in aortic stenosis and clinical implications. Theranostics 2022;12:5189–5203. 35836811 10.7150/thno.71813 PMC9274751

[33] Adhikari R Jung J Shiwakoti S . Capsaicin inhibits aortic valvular interstitial cell calcification via the redox-sensitive NFκB/AKT/ERK1/2 pathway. Biochem Pharmacol 2023;212:115530. 37028459 10.1016/j.bcp.2023.115530

[34] Zheng Y Wen S Jiang S . CircRNA/lncRNA-miRNA-mRNA network and gene landscape in calcific aortic valve disease. BMC Genomics 2023;24:419. 37491214 10.1186/s12864-023-09441-y PMC10367311

[35] Lee SJ Lee IK Jeon JH . Vascular calcification-new insights into its mechanism. Int J Mol Sci 2020;21:2685. 32294899 10.3390/ijms21082685 PMC7216228

[36] Jung C Lichtenauer M Figulla HR . Microparticles in patients undergoing transcatheter aortic valve implantation (TAVI). Heart Vessels 2017;32:458–466. 27488119 10.1007/s00380-016-0885-z PMC5371631

[37] Lugo-Gavidia LM Burger D Matthews VB . Role of microparticles in cardiovascular disease: implications for endothelial dysfunction, thrombosis, and inflammation. Hypertension 2021;77:1825–1844. 33979187 10.1161/HYPERTENSIONAHA.121.16975

[38] Vilcant V Hai O . Left Ventricular Outflow Tract Obstruction. StatPearls. StatPearls Publishing; 2023. http://www.ncbi.nlm.nih.gov/books/NBK470446/ 29262119

[39] Lee HJ Kim HK . Natural history data in symptomatic severe aortic stenosis alerts cardiologists to the dangers of no action. Korean Circ J 2019;49:170–172. 30468037 10.4070/kcj.2018.0344 PMC6351281

[40] Hannoush H Introne WJ Chen MY . Aortic stenosis and vascular calcifications in alkaptonuria. Mol Genet Metab 2012;105:198–202. 22100375 10.1016/j.ymgme.2011.10.017 PMC3276068

[41] Roberts WC Ko JM . Frequency by decades of unicuspid, bicuspid, and tricuspid aortic valves in adults having isolated aortic valve replacement for aortic stenosis, with or without associated aortic regurgitation. Circulation 2005;111:920–925. 15710758 10.1161/01.CIR.0000155623.48408.C5

[42] Otto CM Nishimura RA Bonow RO . 2020 ACC/AHA guideline for the management of patients with valvular heart disease: executive summary: a report of the American College of Cardiology/American Heart Association Joint Committee on Clinical Practice Guidelines. Circulation 2021;143:e35–e71. 33332149 10.1161/CIR.0000000000000932

[43] Lindman BR Bonow RO Otto CM . Current management of calcific aortic stenosis. Circ Res 2013;113:223–237. 23833296 10.1161/CIRCRESAHA.111.300084 PMC4013234

[44] Pujari SH Agasthi P . Aortic Stenosis. StatPearls. StatPearls Publishing; 2023. http://www.ncbi.nlm.nih.gov/books/NBK557628/ 32491560

[45] Zhu F Kaiser Y Boersma E . Aortic valve calcium in relation to subclinical cardiac dysfunction and risk of heart failure. Circ Cardiovasc Imaging 2023;16:e014323. 36880390 10.1161/CIRCIMAGING.122.014323 PMC10026958

[46] Christensen JL Tan S Chung HE . Aortic valve calcification predicts all-cause mortality independent of coronary calcification and severe stenosis. Atherosclerosis 2020;307:16–20. 32702536 10.1016/j.atherosclerosis.2020.06.019 PMC7583087

[47] Chung HE Chen J Ghosalkar D . Aortic valve calcification is associated with future cognitive impairment. J Alzheimers Dis Rep 2021;5:337–343. 34113789 10.3233/ADR-200253 PMC8150250

[48] Fazlinejad A Rohani A Layeghian M . Aortic stenosis: a routine diagnosis with a rare cause. J Cardiovasc Echogr 2016;26:104–105. 28465973 10.4103/2211-4122.187965 PMC5224668

[49] Greco M Robinson JD Eltayeb O . Progressive aortic stenosis in homozygous familial hypercholesterolemia after liver transplant. Pediatrics 2016;138:e20160740. 27940769 10.1542/peds.2016-0740

[50] Narasimhan M Muthu P Ramakrishnan R . Tuberous xanthoma with cardiac failure in a child. J Fam Med Prim Care 2022;11:3290–3292. 10.4103/jfmpc.jfmpc_1416_21 PMC9480728 36119308

[51] Gottschalk BH Blankenstein J Guo L . Ochronosis of mitral valve and coronary arteries. Ann Thorac Surg 2018;106:e19–e20. 29501639 10.1016/j.athoracsur.2018.01.074

[52] Ghadiam H Mungee S . Singleton Merten Syndrome: a rare cause of early onset aortic stenosis. Case Rep Cardiol 2017;2017:8197954. 28321341 10.1155/2017/8197954 PMC5339492

[53] Oxlund CS Hansen H Hansen S . Progressive valvular calcifications with critical aortic stenosis in a 25-year-old woman with end-stage renal disease on haemodialysis: a case report. Eur Heart J Case Rep 2021;5:ytab061. 34345761 10.1093/ehjcr/ytab061 PMC8323062

[54] Taoussi R Khattab H Jadib A . Transient ischemic attack due to multiple spontaneous calcified embolus of the cerebral arteries on a calcified mitral and aortic stenosis. Radiol Case Rep 2022;17:2899–2901. 35733951 10.1016/j.radcr.2022.05.043 PMC9207546

[55] Cacciapuoti F Mauro C Caso I . An unusual cause of iron deficiency anemia in a patient with severe aortic stenosis and heart failure. J Cardiol Cases 2023;27:188–191. 37012914 10.1016/j.jccase.2023.01.002 PMC10066434

[56] Calle-Muller C Rabbani B Khan A . Recurrent ventricular tachycardia from severe aortic stenosis improves after percutaneous aortic balloon valvuloplasty and transcatheter aortic valve replacement. J Cardiol Cases 2014;9:236–238. 30534335 10.1016/j.jccase.2014.03.005 PMC6278675

[57] Kundu A Ogunsua AA Walker J . Bilateral vas deferens calcification in a patient with multi-vessel coronary artery disease and severe aortic stenosis: linking infertility with cardiovascular disease. J Community Hosp Intern Med Perspect 2021;11:376–378. 34234910 10.1080/20009666.2021.1898085 PMC8118445

[58] Lourdusamy D Mupparaju VK Sharif NF . Aortic stenosis and Heyde’s syndrome: a comprehensive review. World J Clin Cases 2021;9:7319–7329. 34616798 10.12998/wjcc.v9.i25.7319 PMC8464459

[59] De Carlini CC Cantù E Erba N . Gastrointestinal bleeding associated to aortic valve stenosis (Heyde’s syndrome): a case series and literature review. Eur Heart J Case Rep 2023;7:ytad412. 37650079 10.1093/ehjcr/ytad412 PMC10464592

[60] Kvaslerud AB Hussain AI Auensen A . Prevalence and prognostic implication of iron deficiency and anaemia in patients with severe aortic stenosis. Open Heart 2018;5:e000901. 30613413 10.1136/openhrt-2018-000901 PMC6307621

[61] Tsuchiya S Matsumoto Y Suzuki H . Transcatheter aortic valve implantation and cognitive function in elderly patients with severe aortic stenosis. EuroIntervention 2020;15:1580. 10.4244/EIJ-D-19-00489 31951203

[62] Bugnicourt JM Bonnaire B Lepage L . [Stroke due to spontaneous calcified cerebral embolus as presenting feature of calcified aortic stenosis]. J Mal Vasc 2008;33:106–109. 18455337 10.1016/j.jmv.2008.02.004

[63] Kurtz CE Otto CM . Aortic stenosis: clinical aspects of diagnosis and management, with 10 illustrative case reports from a 25-year experience. Medicine (Baltimore) 2010;89:349. 21057260 10.1097/MD.0b013e3181fe5648

[64] Singh PK Jangpangi G . Visible pulsus parvus et tardus in patient of aortic stenosis. BMJ Case Rep 2017;2017:bcr2017221034. 10.1136/bcr-2017-221034 PMC5535114 28679515

[65] Stanger D Wan D Moghaddam N . Insonation versus auscultation in valvular disorders: is aortic stenosis the exception? A systematic review. Ann Glob Health 2019;85:104. 31298821 10.5334/aogh.2489 PMC6634326

[66] Boeuf MC Rohel G Lamour G . [Diagnosis of a systolic murmur among young asymptomatic patient: an assessment of professional practices for the expertise in military medicine]. Ann Cardiol Angeiol (Paris) 2015;64:352–361. 26482624 10.1016/j.ancard.2015.09.042

[67] Abraham B Farina JM Fath A . The impact of moderate aortic stenosis in acute myocardial infarction: a multicenter retrospective study. Catheter Cardiovasc Interv 2023;102:159–165. 37146200 10.1002/ccd.30676

[68] Gottlieb M Long B Koyfman A . Evaluation and management of aortic stenosis for the emergency clinician: an evidence-based review of the literature. J Emerg Med 2018;55:34–41. 29525246 10.1016/j.jemermed.2018.01.026

[69] Sorrentino SA . Radiopaedia, 2023. Aortic valve stenosis. Accessed 11 September 2023. https://radiopaedia.org/articles/aortic-valve-stenosis

[70] Lefort B Cheyssac E Soulé N . Auscultation while standing: a basic and reliable method to rule out a pathologic heart murmur in children. Ann Fam Med 2017;15:523–528. 29133490 10.1370/afm.2105 PMC5683863

[71] Klocko DJ Hanifin C . Cardiac auscultation: using physiologic maneuvers to further identify heart murmurs. JAAPA 2019;32:21–25. 10.1097/01.JAA.0000604856.33701.ad 31714345

[72] Srivastav S Jamil RT Zeltser R . Valsalva Maneuver. StatPearls. StatPearls Publishing; 2023. http://www.ncbi.nlm.nih.gov/books/NBK537248/ 30725933

[73] Rana M . Aortic valve stenosis: diagnostic approaches and recommendations of the 2021 ESC/EACTS guidelines for the management of valvular heart disease – a review of the literature. Cardiol Cardiovasc Med 2022;6:315–324. 36035621 10.26502/fccm.92920267 PMC9410571

[74] Cohen-Shelly M Attia ZI Friedman PA . Electrocardiogram screening for aortic valve stenosis using artificial intelligence. Eur Heart J 2021;42:2885–2896. 33748852 10.1093/eurheartj/ehab153

[75] Mino T Kimura S Kitaura A . Can left ventricular hypertrophy on electrocardiography detect severe aortic valve stenosis? PLoS One 2020;15:e0241591. 33147268 10.1371/journal.pone.0241591 PMC7641401

[76] Ring L Shah BN Bhattacharyya S . Echocardiographic assessment of aortic stenosis: a practical guideline from the British Society of Echocardiography. Echo Res Pract 2021;8:G19–G59. 33709955 10.1530/ERP-20-0035 PMC8115410

[77] Cartlidge TR Bing R Kwiecinski J . Contrast-enhanced computed tomography assessment of aortic stenosis. Heart 2021;107:1905–1911. 33514522 10.1136/heartjnl-2020-318556 PMC8600609

[78] Pawade T Sheth T Guzzetti E . Why and how to measure aortic valve calcification in patients with aortic stenosis. JACC Cardiovasc Imaging 2019;12:1835–1848. 31488252 10.1016/j.jcmg.2019.01.045

[79] Blanke P Weir-McCall JR Achenbach S . Computed tomography imaging in the context of transcatheter aortic valve implantation (TAVI)/transcatheter aortic valve replacement (TAVR): an expert consensus document of the society of cardiovascular computed tomography. JACC Cardiovasc Imaging 2019;12:1–24. 30621986 10.1016/j.jcmg.2018.12.003

[80] Guglielmo M Rovera C Rabbat MG . The role of cardiac magnetic resonance in aortic stenosis and regurgitation. J Cardiovasc Dev Dis 2022;9:108. 35448084 10.3390/jcdd9040108 PMC9030119

[81] Bohbot Y Renard C Manrique A . Usefulness of cardiac magnetic resonance imaging in aortic stenosis. Circ Cardiovasc Imaging 2020;13:e010356. 32370617 10.1161/CIRCIMAGING.119.010356

[82] Nishimura RA Otto CM Bonow RO . 2017 AHA/ACC focused update of the 2014 AHA/ACC guideline for the management of patients with valvular heart disease: a report of the American College of Cardiology/American Heart Association Task Force on Clinical Practice Guidelines. Circulation 2017;135:e1159–e1195. 28298458 10.1161/CIR.0000000000000503

[83] Baumgartner H Hung J Bermejo J . Echocardiographic assessment of valve stenosis: EAE/ASE recommendations for clinical practice. J Am Soc Echocardiogr 2009;22:1–23; quiz 101–2. 19130998 10.1016/j.echo.2008.11.029

[84] Donato M Ferri N Lupo MG . Current evidence and future perspectives on pharmacological treatment of calcific aortic valve stenosis. Int J Mol Sci 2020;21:8263. 33158204 10.3390/ijms21218263 PMC7663524

[85] Shah SM Shah J Lakey SM . Pathophysiology, emerging techniques for the assessment and novel treatment of aortic stenosis. Open Heart 2023;10:e002244. 36963766 10.1136/openhrt-2022-002244 PMC10040005

[86] Boskovski MT Gleason TG . Current therapeutic options in aortic stenosis. Circ Res 2021;128:1398–1417. 33914604 10.1161/CIRCRESAHA.121.318040

[87] Ontario Health (Quality) . Transcatheter aortic valve implantation in patients with severe, symptomatic aortic valve stenosis at intermediate surgical risk: a health technology assessment. Ont Health Technol Assess Ser 2020;20:1–121. PMC7080451 32194880

[88] Rajput FA Zeltser R: . Aortic Valve Replacement. StatPearls. StatPearls Publishing; 2023. http://www.ncbi.nlm.nih.gov/books/NBK537136/ 30725821

[89] Goyal A Jain H Verma A . The role of renal denervation in cardiology and beyond: an updated comprehensive review and future directives. Curr Prob Cardiol 2024;49:102196. 10.1016/j.cpcardiol.2023.102196 37952794

[90] Schäfer A Fraccarollo D Werner L . Guanylyl cyclase activator ataciguat improves vascular function and reduces platelet activation in heart failure. Pharmacol Res 2010;62:432–438. 20600916 10.1016/j.phrs.2010.06.008

[91] Sandner P Zimmer DP Milne GT . Soluble guanylate cyclase stimulators and activators. Handb Exp Pharmacol 2021;264:355–394. 30689085 10.1007/164_2018_197

[92] Wang W Liu C Cong H . Hypothesis: new PCSK9 inhibitors may impact calcific aortic valve disease. J Cardiovasc Pharmacol Ther 2017;22:56–64. 27246356 10.1177/1074248416651721

[93] Pawade TA Doris MK Bing R . Effect of denosumab or alendronic acid on the progression of aortic stenosis: a double-blind randomized controlled trial. Circulation 2021;143:2418–2427. 33913339 10.1161/CIRCULATIONAHA.121.053708 PMC8212878

[94] Li S Luo Z Su S . Targeted inhibition of PTPN22 is a novel approach to alleviate osteogenic responses in aortic valve interstitial cells and aortic valve lesions in mice. BMC Med 2023;21:252. 37443055 10.1186/s12916-023-02888-6 PMC10347738

[95] Lei Y Grover A Sinha A . Efficacy of reversal of aortic calcification by chelating agents. Calcif Tissue Int 2013;93:426–435. 23963635 10.1007/s00223-013-9780-0 PMC3809012

[96] Goyal A Changez MIK Tariq MD . Efficacy and outcomes of Bempedoic acid versus placebo in patients with statin-intolerance: a pilot systematic review and meta-analysis of randomized controlled trials. Curr Probl Cardiol 2024;49:102236. 38043880 10.1016/j.cpcardiol.2023.102236

[97] Tzolos E Kwiecinski J Berman D . Latest advances in multimodality imaging of aortic stenosis. J Nucl Med 2022;63:353–358. 34887339 10.2967/jnumed.121.262304 PMC8978201

[98] Doris MK Otaki Y Krishnan SK . Optimization of reconstruction and quantification of motion-corrected coronary PET-CT. J Nucl Cardiol 2020;27:494–504. 29948889 10.1007/s12350-018-1317-5 PMC6289874

[99] Lohrke J Siebeneicher H Berger M . 18F-GP1, a novel PET tracer designed for high-sensitivity, low-background detection of thrombi. J Nucl Med 2017;58:1094–1099. 28302764 10.2967/jnumed.116.188896

[100] Tarkin JM Joshi FR Evans NR . Detection of atherosclerotic inflammation by 68Ga-DOTATATE PET compared to [18F]FDG PET imaging. J Am Coll Cardiol 2017;69:1774–1791. 28385306 10.1016/j.jacc.2017.01.060 PMC5381358

[101] Moncla LHM Briend M Bossé Y . Calcific aortic valve disease: mechanisms, prevention and treatment. Nat Rev Cardiol 2023;20:546–559. 36829083 10.1038/s41569-023-00845-7

[102] Koos R Brandenburg V Mahnken AH . Association of fetuin-A levels with the progression of aortic valve calcification in non-dialyzed patients. Eur Heart J 2009;30:2054–2061. 19429630 10.1093/eurheartj/ehp158

[103] Pasch A Block GA Bachtler M . Blood calcification propensity, cardiovascular events, and survival in patients receiving hemodialysis in the EVOLVE trial. Clin J Am Soc Nephrol 2017;12:315–322. 27940458 10.2215/CJN.04720416 PMC5293330

[104] Yadgir S Johnson CO Aboyans V . Global, regional, and national burden of calcific aortic valve and degenerative mitral valve diseases, 1990-2017. Circulation 2020;141:1670–1680. 32223336 10.1161/CIRCULATIONAHA.119.043391

[105] Otto CM Prendergast B . Aortic-valve stenosis--from patients at risk to severe valve obstruction. N Engl J Med 2014;371:744–756. 25140960 10.1056/NEJMra1313875

[106] Plunde O Bäck M . Fatty acids and aortic valve stenosis. Kardiol Pol 2021;79:614–621. 34002847 10.33963/KP.a2021.0003

[107] Lindman BR Alexander KP O’Gara PT . Futility, benefit, and transcatheter aortic valve replacement. JACC Cardiovasc Interv 2014;7:707–716. 24954571 10.1016/j.jcin.2014.01.167 PMC4322002

[108] Reynolds MR Magnuson EA Wang K . Health-related quality of life after transcatheter or surgical aortic valve replacement in high-risk patients with severe aortic stenosis: results from the PARTNER (Placement of AoRTic TraNscathetER Valve) Trial (Cohort A). J Am Coll Cardiol 2012;60:548–558. 22818074 10.1016/j.jacc.2012.03.075

[109] Tamuleviciute-Prasciene E Drulyte K Jurenaite G . Frailty and exercise training: how to provide best care after cardiac surgery or intervention for elder patients with valvular heart disease. BioMed Res Int 2018;2018:9849475. 30302342 10.1155/2018/9849475 PMC6158962

[110] Généreux P Pibarot P Redfors B . Evolution and prognostic impact of cardiac damage after aortic valve replacement. J Am Coll Cardiol 2022;80:783–800. 35595203 10.1016/j.jacc.2022.05.006

[111] Bogyi M Schernthaner RE Loewe C . Subclinical leaflet thrombosis after transcatheter aortic valve replacement: a meta-analysis. JACC Cardiovasc Interv 2021;14:2643–2656. 34949391 10.1016/j.jcin.2021.09.019

[112] Del Val D Panagides V Mestres CA . Infective endocarditis after transcatheter aortic valve replacement: JACC state-of-the-art review. J Am Coll Cardiol 2023;81:394–412. 36697140 10.1016/j.jacc.2022.11.028

[113] Bombace S Meucci MC Fortuni F . Beyond aortic stenosis: addressing the challenges of multivalvular disease assessment. Diagnostics (Basel) 2023;13:2102. 37370999 10.3390/diagnostics13122102 PMC10297357

[114] Anwaruddin S Saybolt MD . Improving quality and outcomes in TAVR: turning up the volume? J Am Coll Cardiol 2019;73:441–443. 30704576 10.1016/j.jacc.2018.09.093

[115] Lindman BR Sukul D Dweck MR . Evaluating medical therapy for calcific aortic stenosis: JACC state-of-the-art review. J Am Coll Cardiol 2021;78:2354–2376. 34857095 10.1016/j.jacc.2021.09.1367 PMC8647810

[116] Kapelouzou A Geronikolou S Lidoriki I . Tissue and serum biomarkers in degenerative aortic stenosis-insights into pathogenesis, prevention and therapy. Biology 2023;12:347. 36979039 10.3390/biology12030347 PMC10045285

[117] Baran J Niewiara Ł Podolec J . Serum and vascular stiffness biomarkers associated with the severity of degenerative aortic valve stenosis and cardiovascular outcomes. J Cardiovasc Dev Dis 2022;9:193. 35735822 10.3390/jcdd9060193 PMC9225443

[118] Peeters FECM Dudink EAMP Weijs B . Biomarkers associated with aortic valve calcification: should we focus on sex specific processes? Front Cell Dev Biol 2020;8:604. 32754594 10.3389/fcell.2020.00604 PMC7366171

[119] Vidula MK Orlenko A Zhao L . Plasma biomarkers associated with adverse outcomes in patients with calcific aortic stenosis. Eur J Heart Fail 2021;23:2021–2032. 34632675 10.1002/ejhf.2361

[120] Liu F Qu B Wang L . Effect of selective sleep deprivation on heart rate variability in post-90s healthy volunteers. Math Biosci Eng 2022;19:13851–13860. 36654070 10.3934/mbe.2022645

[121] Schlotter F de Freitas RCC Rogers MA . ApoC-III is a novel inducer of calcification in human aortic valves. J Biol Chem 2021;296:100193. 33334888 10.1074/jbc.RA120.015700 PMC7948477

[122] Yalta K Palabiyik O Gurdogan M . Serum copeptin might improve risk stratification and management of aortic valve stenosis: a review of pathophysiological insights and practical implications. Ther Adv Cardiovasc Dis 2019;13:1753944719826420. 30803406 10.1177/1753944719826420 PMC6376527

